# Synthesis of l-Ascorbic Acid Lactone Derivatives

**DOI:** 10.1007/s13659-014-0022-6

**Published:** 2014-05-21

**Authors:** Li-Dong Shao, Ya-Nan Wu, Jun Xu, Juan He, Yu Zhao, Li-Yan Peng, Yan Li, Yu-Rong Yang, Cheng-Feng Xia, Qin-Shi Zhao

**Affiliations:** 1 State Key Laboratory of Phytochemistry and Plant Resources in West China, Kunming Institute of Botany, Chinese Academy of Sciences, Kunming, 650201 China; 2 Graduate School of the Chinese Academy of Sciences, University of Chinese Academy of Sciences, Beijing, 100049 China

**Keywords:** l-Ascorbic acid lactone, Cytotoxicity, Focused library

## Abstract

A small focused library which comprised of l-AA lactone derivatives was built with a facile method. This reported method was optimized by modifying the acidity of the solvent. As a result, 12 l-AA lactones were synthesized. Among these lactones, lactones **8**–**12** were new compounds. The cytotoxicity of these synthetic compounds were investigated.

## Introduction

l-Ascorbic acid (l-AA), one form of vitamin C, plays an important role in both plant and animal physiology. The foremost biologically functions of l-AA are centred around the antioxidant properties. Considerable evidence has been accruing in the last two decades about the importance of l-AA not only in protecting the plant from oxidative stress, but also in protecting mammals from various chronic diseases that have their origins in oxidative stress [1]. Derivatives of l-AA were found showing wide range of bioactivities including antiviral [2–5], cytotoxicity [6], inhibitory activities against tyrosinase-catalyzed melanin formation [7], increasing skin permeability [8, 9], and neurotropic activity [10]. Among them, octanoyl-6-*O*-ascorbic acid could enhance the solubility of many poorly water soluble drugs [11]. Because of these properties, l-AA derivatives were applicable in cosmetics and medicine [12, 13].

**Fig. 1:**
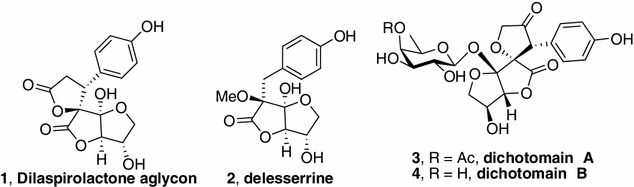
Natural l-AA lactone derivatives

Many bioactive l-AA derivatives were found in nature [14–16]. For example, bioactive-oriented isolation of dilaspirolactone aglycon (**1**) and delesserrine (**2**) (Fig. 1) from Delesseriaceae family were reported [17, 18]. Our research group are interested in fern plants for a long time. A lot of species were systematically studied towards chemical components and their bioactivities [19–23], which led to the isolate of dichotomains A and B (**3**, **4**) (Fig. 1), two l-AA derivatives, from *Dicranopteris dichotoma*. And dichotomain B (**4**) was confirmed as a weak HIV-1 inhibitor [24]. These compounds with a fragment of l-AA lactone showed different bioactivities. Attracted by this difference and the unique structure of l-AA derivatives, we would like to build a small focused library of l-AA lactone derivatives to explore their bioactivities.

## Results and Discussion

**Scheme 1:**
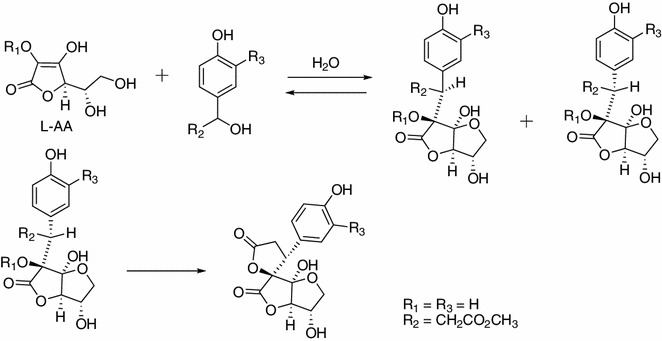
A synthetic route to l-AA lactone derivatives reported by Poss et al

**Fig. 2:**
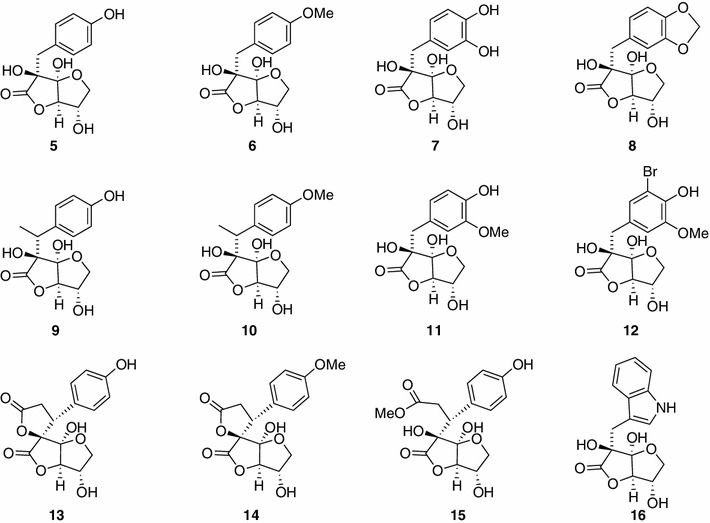
Synthetic l-AA lactone derivatives

Tang et al. [25] reported a short total synthesis of l-AA lactone compounds leucodrin and leudrin through a organocatalystic 1,4-conjugate addition of l-AA to *α*,*β*-unsaturated aldehydes. Although it effectively synthesized 5/5/5 spirodilactone l-AA derivatives, it could not access to other l-AA lactone derivatives. Poss et al. [26] reported that treating l-AA with different 4-hydoxy benzyl alcohols in hot water resulted in l-AA lactone derivatives (scheme 1). With this method, we obtained some l-AA lactone derivatives (**5**, **7**, **13**, **14**, **15**, Fig. 2), but a number of l-AA lactone derivatives (**6**, **8**, **9**, **10**, **11**, **12**, **16**, Fig. 2) could not formed by using this methods. The failure probably was caused by acidity of the solvent [25, 27], so, we modified the condition by applying the phosphate-citrate buffer solution (PH 5.0) as solvent. As a result, compounds **6**, **8**, **9**, **10**, **11**, **12**, **16** were successfully synthesized.

**Scheme 2:**
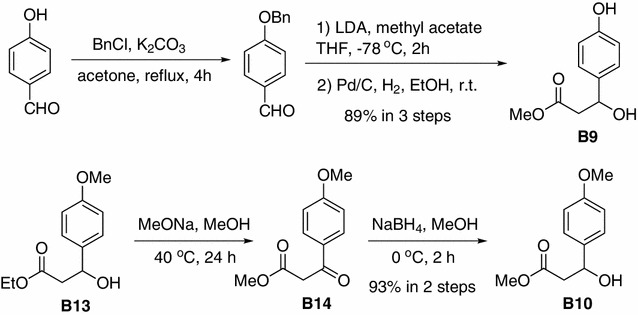
Synthetic route to compounds **B9** and **B10**

All 4-hydroxy benzyl alcohols were synthesized by reduction of corresponding aldehydes with NaBH_4_ except **B9** and **B10** (Table 1). Without following the Ref. [26], 4-hydroxy benzaldehyde was protected with Bn group, and then reacted with methyl acetate through an aldol condensation. At last, removal of Bn group gave **B9** in 89 % yield. It is interesting that **B13** could not react with l-AA to yield lactone compound. However, it worked with methyl ether in stead of ethyl ether. An air oxidative product **B14** was detected in methanolysis reaction of **B13**, which was reducted by NaBH_4_ to afford **B10** (Scheme 2).

**Table 1:** Synthesis of l-AA lactone derivatives

Entry	4-hydroxy benzyl alcohol	Condition	Product (yields)
1		H_2_0, 50 °C, 50 h	**5** (85 %)
2		buffer, 40 °C, 72 h	**6** (53 %)
3		H_2_0, 50 °C, 72 h	**7** (35 %)
4		buffer, 60 °C, 36 h	**8** (72 %)
5		buffer, 60 °C, 60 h	**9** (65 %) 10:1 of two benzylic siomers
6		buffer, 60 °C, 72 h	**10** (42 %) 3.4:1 of two benzylic siomers
7		buffer, 40 °C, 48 h	**11** (78 %)
8		buffer, 60 °C, 72 h	**12** (58 %)
9		H_2_0, 50 °C, 72 h	**13** (35 %) **15** (40 %)
10		H_2_0, 50 °C, 72 h	**14** (30 %)
11		buffer/EtOH, r.t, 8 h	**16** (57 %)
12		H_2_O, 50 °C, 72 h	nr
		buffer, 60 °C, 72 h	
		buffer/EtOH, r.t., 72 h

With all designed 4-hydoxy benzyl alcohols in hand, we built a small focused library which contained l-AA lactone drivatives **5**–**16**. We found that 4-hydoxy benzyl alcohols like **B1**, **B4**, **B5**, **B7**, and **B11** with good water solubility could react well with l-AA to give lactone derivatives in good yield except **B3**. This might be that two phenolic hydroxyl groups in **B3** made it be easily oxidized by air. **B12** could not react with l-AA in all conditions applied in this article, probably because the reactivity of lone pair electron at S atom of **B12** was lower than that of phenol hydroxyl. So, it could not react like other 4-hydoxy benzyl alcohols. Furthermore, all l-AA lactones were obtained as a single compound except lactones **9** and **10** which were mixtures of two isomers at benzylic position. ^1^H NMR indicated their ratio is about 10:1 for **9** and 3.4:1 for **10**, and we found that one isomer of **9** and **10** were unstable and could transform from semi-ketal into ketone at C3 position at room temperature or under NMR condition spontaneously.

All synthetic L-AA lactone derivatives were evaluated on five human tumor cell lines, including HL-60, SMMC-7721, A-549, MCF-7 and SW480, using 3-(4,5-dimethylthiazol-2-yl)-2,5-diphenyltetrazolium bromide (MTT) method (Table 2). Anticancer drug cisplatin (DDP) was used as the positive control. To our disappointment, none of these compounds showed cytotoxicity.

**Table 2:** In vitro anti-tumor assay of the synthetic compounds

Entry	IC_50_(*μ*M)				
	HL-60	SMMC-7721	A-549	MCF-7	SW480
5	>40	>40	>40	>40	>40
6	>40	>40	>40	>40	>40
7	>40	>40	>40	>40	>40
8	>40	>40	>40	>40	>40
9	>40	>40	>40	>40	>40
10	>40	>40	>40	>40	>40
11	>40	>40	>40	>40	>40
12	>40	>40	>40	>40	>40
13	>40	>40	>40	>40	>40
14	>40	>40	>40	>40	>40
15	>40	>40	>40	>40	>40
16	>40	>40	>40	>40	>40
DPP	1.05	6.76	6.01	15.38	16.31
Taxol	<0.008	<0.008	<0.008	<0.008	<0.008

## Experiment Section

### General Experimental Procedures

HRESIMS were performed on a Agilent 6540 Q-TOF. ^1^H and ^13^C NMR spectra were recorded on Bruker Avance III-400 and Bruker Avance III-600 MHz spectrometers. Chemical shifts (*δ*) were expressed in *ppm* with reference to the TMS resonance. Column chromatography was performed using Silica gel [(200–300) mesh, Qingdao Marine Chemical, Inc, Qingdao, China]. Reactions were monitored by TLC and spots were visualized by heating the silica gel plates sprayed with 10 % H_2_SO_4_ in EtOH.

### Synthesis of 4-Hydroxy Benzyl Alcohols (**B1**–**B8**, **B11**–**B12**)

4-Hydroxy benzyl alcohols **B1**–**B8**, **B11**–**B12** were synthesized by reduction of corresponding commercial available aldehydes with NaBH_4_ in MeOH at 0 °C for 1–4 h.

**B3**: Brown foam, 90 % yields, ^1^H NMR (400 MHz, D_2_O) *δ* 6.50–6.56 (m, 3H), 4.34 (s, 2H).

**B5**: Light yellow oil, 96 % yields, ^1^H NMR (400 MHz, acetone-D6) *δ* 8.29 (s, 1H), 7.20 (d, *J* = 8.4 Hz, 1H), 6.77 (d, *J* = 8.4 Hz, 1H), 4.75 (m, 1H), 4.04 (d, *J* = 4 Hz, 1H), 1.36 (d, *J* = 6.4 Hz, 3H).

**B7**: White solid, 92 % yields, ^1^H NMR (400 MHz, CDCl_3_) *δ* 9.72 (s, 1H), 7.57 (d, *J* = 1.6 Hz, 1H), 7.30 (d, *J* = 1.6 Hz, 1H), 6.48 (s, 1H), 3.91 (s, 3H).

**B8**: White solid, 82 % yields, ^1^H NMR (400 MHz, CDCl_3_) *δ* 7.02 (d, *J* = 1.0 Hz, 1H), 6.79 (d, *J* = 1.0 Hz, 1H), 5.86 (br. s, 1H), 4.53 (s, 2H), 3.85 (s, 3H).

### Synthesis of 4-Hydroxy Benzyl Alcohols (**B9**, **B10**)

**B9**: To a solution of 4-hydroxy benzldehyde (12.2 g, 0.1 mol) in 100 mL acetone, K_2_CO_3_ (20.7 g, 0.15 mol) and BnCl (12.6 mL, 0.11 mol) was added, which was refluxed for 4 h. Cooled to room temperature, 20 mL ice-water was added and extracted with EtOAc (3 × 100 mL). The organic layers were combined and washed by brine (3 × 50 mL), dried over Na_2_SO_4_ (s), then evaporated the solvent under the reduced pressure to give 4-benzyloxy benzldehyde as a light yellow solid (21 g, 99 %). This compound was used in next step without further purification.

To a solution of freshly distilled diisopropylamine (14 mL, 0.12 mol) in 100 mL dry THF at −78 °C was added *n*-BuLi (50 mL of 2 M in hexane, 0.1 mol) and stirred for 15 min. Freshly distilled methyl acetate (8.6 mL, 0.11 mol) was added. The reaction stirred for 1 h at −78 °C, and 4-benzyloxyl benzldehyde (21 g, 0.1 mol) in 100 mL dry THF was added. After 1 h at −78 °C, the reaction was quenched with saturated NH_4_Cl aqueous solution (50 mL), warmed to room temperature, and stirred for an additional 6 h. The solution was extracted with EtOAc (3 × 100 mL), and the organic layers were combined and washed by brine (3 × 50 mL), dried over Na_2_SO_4_ (s), evaporated the solvent under the reduced pressure to give crude product as a yellow solid, which was purified by flash chromatography with petroleum ether/EtOAc (20/1) to give methyl 3-(4-(benzyloxy)phenyl)-3-hydroxypropanoate 26 g (91 %) as a white solid. ^1^H NMR (400 MHz, CDCl_3_) *δ* 7.29 (m, 7H), 6.96 (dd, *J* = 1.9, 8.0 Hz, 2H), 5.08 (dd, *J* = 3.7, 9.2 Hz, 1H), 5.06 (s, 2H), 3.72 (s, 3H), 2.66–2.80 (m, 2H).

To a solution of methyl 3-(4-(benzyloxy)phenyl)-3-hydroxypropanoate (5 g, 17.48 mmol) in 50 mL EtOH, 500 mg 10 % Pd/C was added, which then stirred under H_2_ atmosphere overnight at room temperature. The reaction mixture was passed through a short pad of Celite to remove Pd/C and evaporated the solvent under the reduced pressure to give 3.4 g (100 %) of **B9** as a white foam. ^1^H NMR (400 MHz, CDCl_3_) *δ* 7.22 (d, *J* = 8.5 Hz, 2H), 6.78 (d, *J* = 8.5 Hz, 2H), 5.07 (dd, *J* = 3.7, 9.2 Hz, 1H), 3.72 (s, 3H), 2.66–2.81 (m, 2H).

**B10**: To a solution of **B13** (10 g, 44.6 mmol) in anhydrous MeOH, MeONa (240 mg, 4.46 mmol) was added. The mixture was stirred at 40 °C for 24 h. Evaporated the solvent under the reduced pressure, the residue was dissolved in 100 mL EtOAc, washed by water (3 × 50 mL), brine (3 × 50 mL), dried over Na_2_SO_4_ (s). Evaporated the solvent under the reduced pressure to give 9.28 g (100 %) of **B14** as a light yellow oil. ^1^H NMR (400 MHz, CDCl_3_) *δ* 7.91 (d, *J* = 8.8 Hz, 2H), 6.93 (d, *J* = 8.8 Hz, 2H), 3.95 (s, 2H), 3.86 (s, 3H), 3.73 (s, 3H).

To a solution of **B14** (5 g, 24 mmol) in 30 mL MeOH at 0 °C was added NaBH_4_ (1.82 g, 48 mmol), the mixture was stirred at 0 °C for 2 h. The reaction was quenched with saturated NH_4_Cl aqueous solution (5 mL) at 0 °C. The resulting mixture was extracted with EtOAc (3 × 30 mL), and the organic layers were combined and washed by brine (3 × 50 mL), dried over Na_2_SO_4_ (s). Evaporated the solvent under the reduced pressure to give crude product as a colorless oil, and the crude product was purified by flash chromatography with petroleum ether/EtOAc (20/1) to give 4.7 g (93 %) of **B10** as a colorless oil. ^1^H NMR (400 MHz, CDCl_3_) *δ* 7.30 (dd, *J* = 2.8, 8.6 Hz, 2H), 6.90 (dd, *J* = 2.8, 8.6 Hz, 2H), 5.08 (dd, *J* = 3.7, 9.3 Hz, 1H), 3.80 (s, 3H), 3.71 (s, 3H), 2.65–2.80 (m, 2H).

### General Procedure for the Preparation of l-AA Lactone Derivatives (5–16)

Method A [26] for lactones **5**, **7**, **13**, **14**, **15**: To l-AA (3 eq.) in 2 mL water was added corresponding alcohol **B1** or **B3** or **B9** or **B10** (0.5 mmol, 1 eq.), and the solution stirred at 50 °C for 72 h. The reaction was evaporated under reduced pressure and the residue was purified by column chromatography with DCM/MeOH (50/1–20/1) to afford lactone **5** or **7** or **13**, **15** or **14**, respectively.

**5** (85 %), white foam: ^1^H NMR (600 MHz, acetone-*d*_6_) *δ* 8.31 (s, 1H), 7.14 (d, *J* = 8.5 Hz, 2H), 6.73 (d, *J* = 8.5 Hz, 2H), 5.86 (s, 1H), 4.66 (s, 1H), 4.45 (s, 1H), 4.30 (s, 1H), 4.09 (dd, *J* = 9.7, 5.5 Hz, 1H), 4.00 (dd, *J* = 9.7, 3.1 Hz, 1H), 3.77 (s, 1H), 3.09 (d, *J* = 13.5 Hz, 1H), 2.92 (d, *J* = 13.5 Hz, 1H). ^13^C NMR (150 MHz, acetone-*d*_6_) *δ* 175.88, 157.41, 132.83, 125.75, 115.60, 108.31, 86.97, 80.77, 75.51, 75.45, 55.05, 40.62. HRESIMS *m/z* 305.0636 (calcd for C_13_H_14_O_7_ [M + Na]^+^, 305.0632).

**7** (35 %), yellow oil: ^1^H NMR (600 MHz, acetone-*d*_6_) *δ* 6.81 (d, *J* = 2.0 Hz, 1H), 6.70 (d, *J* = 8.1 Hz, 1H), 6.65 (dd, *J* = 8.1, 2.0 Hz, 1H), 5.85 (s, 1H), 4.66 (s, 1H), 4.42 (s, 1H), 4.30 (s, 1H), 4.09 (dd, *J* = 9.7, 5.5 Hz, 1H), 3.99 (dd, *J* = 9.7, 3.2 Hz, 1H), 3.04 (d, *J* = 13.4 Hz, 1H), 2.87 (s, 1H), 2.85 (d, *J* = 13.4 Hz, 1H). ^13^C NMR (150 MHz, acetone-*d*_6_) *δ* 175.95, 145.25, 145.17, 126.45, 123.30, 118.81, 115.60, 108.34, 86.97, 80.68, 75.49, 75.47, 40.90. HRESIMS *m/z* 333.0377 (calcd for C_13_H_14_O_8_ [M + Na]^+^, 333.0383).

**13** (35 %), white foam: ^1^H NMR (600 MHz, acetone-*d*_6_) *δ* 8.67 (s, 1H), 7.29 (d, *J* = 8.7 Hz, 2H), 6.86 (d, *J* = 8.7 Hz, 2H), 6.56 (s, 1H), 4.79 (s, 1H), 4.25–4.40 (m, 2H), 4.21 (dd, *J* = 9.7, 6.0 Hz, 1H), 4.02 (dd, *J* = 9.7, 3.7 Hz, 1H), 3.90 (s, 1H), 3.17 (dd, *J* = 17.3, 13.2 Hz, 1H), 2.90 (dd, *J* = 17.3, 8.5 Hz, 1H). ^13^C NMR (150 MHz, acetone-*d*_6_) *δ* 174.37, 172.06, 158.70, 130.91, 124.09, 116.48, 106.38, 90.12, 88.98, 76.19, 74.81, 45.66, 33.97. HRESIMS *m/z* 321.0607 (calcd for C_15_H_14_O_8_ [M–H]^−^, 321.0616).

**15** (40 %), light yellow oil: ^1^H NMR (600 MHz, acetone-*d*_6_) *δ* 8.46 (s, 1H), 7.25 (d, *J* = 9.0 Hz, 2H), 6.74 (d, *J* = 9.0 Hz, 2H), 5.78 (s, 1H), 4.72 (s, 1H), 4.26 (m, 1H), 4.02 (m, 2H), 3.62 (m, 1H), 3.47 (s, 3H), 3.29 (d, *J* = 1.5 Hz, 2H). HRESIMS *m/z* 377.0845 (calcd for C_16_H_18_O_9_ [M + Na]^+^, 377.0843).

**14** (30 %), white foam: ^1^H NMR (600 MHz, acetone-*d*_6_) *δ* 8.67 (s, 1H), 7.38 (d, *J* = 9 Hz, 2H), 6.96 (d, *J* = 9 Hz, 2H), 6.56 (s, 1H), 4.79 (s, 1H), 4.25–4.40 (m, 2H), 4.21 (dd, *J* = 9.7, 6.0 Hz, 1H), 4.02 (dd, *J* = 9.7, 3.7 Hz, 1H), 3.90 (s, 1H), 3.81 (s, 3H), 3.17 (dd, *J* = 17.3, 13.2 Hz, 1H), 2.90 (dd, *J* = 17.3, 8.5 Hz, 2H). ^13^C NMR (150 MHz, acetone-*d*_6_) *δ* 174.32, 171.98, 160.88, 130.87, 125.34, 115.02, 106.38, 90.05, 88.95, 76.22, 74.78, 55.62, 45.57, 33.98. HRESIMS *m/z* 359.0740 (calcd for C_16_H_16_O_8_ [M + Na]^+^, 359.0737).

Method B for lactones **6**, **8**, **9**, **10**, **11**, **12**: To l-AA (4 eq.) in 4 mL phosphate-citrate buffer (*p*H = 5.0) was added corresponding alcohol **B2** or **B4** or **B5** or **B6** or **B7** or **B8** (1 mmol, 1 eq.), and the solution stirred at 40–60 °C for 36–72 h. The reaction was evaporated under reduced pressure and the residue was purified by colum chromatography with DCM/MeOH (50/1–20/1) to afford lactone **6** or **8** or **9** or **10** or **11** or **12**, respectively.

**6** (53 %), yellow foam: ^1^H NMR (600 MHz, acetone-*d*_6_) *δ* 7.22 (d, *J* = 8.7 Hz, 2H), 6.82 (d, *J* = 8.7 Hz, 2H), 5.90 (s, 1H), 4.68 (s, 1H), 4.50 (s, 1H), 4.31 (dd, *J* = 5.3, 3.2 Hz, 1H), 4.09 (dd, *J* = 9.7, 5.5 Hz, 1H), 4.01 (dd, *J* = 9.7, 3.1 Hz, 1H), 3.82 (s, 1H), 3.77 (s, 3H), 3.11 (d, *J* = 13.6 Hz, 1H), 2.96 (d, *J* = 13.6 Hz, 1H). ^13^C NMR (150 MHz, acetone-*d*_6_) *δ* 175.74, 159.76, 132.78, 127.07, 114.09, 108.30, 87.03, 80.64, 75.57, 75.44, 55.42, 40.51. HRESIMS *m/z* 319.0788 (calcd for C_14_H_16_O_7_ [M + Na]^+^, 319.0787).

**8** (72 %), white foam: ^1^H NMR (600 MHz, acetone-*d*_6_) *δ* 6.83 (d, *J* = 1.4 Hz, 1H), 6.76 (dt, *J* = 16.3, 4.7 Hz, 2H), 5.96 (d, *J* = 1.7 Hz, 2H), 5.90 (s, 1H), 4.71 (s, 1H), 4.56 (s, 1H), 4.34 (dd, *J* = 4.6, 3.2 Hz, 1H), 4.11 (dd, *J* = 9.7, 5.5 Hz, 1H), 4.03 (m, overlaped, 2H), 3.08 (d, *J* = 13.7 Hz, 1H), 2.96 (d, *J* = 13.7 Hz, 1H). ^13^C NMR (150 MHz, acetone-*d*_6_) *δ* 175.49, 148.05, 147.55, 129.05, 124.87, 111.96, 108.47, 108.31, 101.88, 87.10, 80.48, 75.70, 75.41, 40.91. HRESIMS *m/z* 333.0586 (calcd for C_14_H_14_O_8_ [M + Na]^+^, 333.0581).

**9** (65 %), colorless oil: main isomer: ^1^H NMR (600 MHz, acetone-*d*_6_) *δ* 8.35 (s, 1H), 7.17 (d, *J* = 8.8 Hz, 2H), 6.74 (d, *J* = 8.8 Hz, 2H), 5.71 (s, 1H), 4.57 (d, *J* = 3.7 Hz, 1H), 4.38 (s, 1H), 4.23 (d, *J* = 2.3 Hz, 1H), 4.04 (dd, *J* = 9.7, 5.4 Hz, 1H), 3.95 (dd, *J* = 9.7, 2.9 Hz, 1H), 3.34 (q, *J* = 7.2 Hz, 1H), 3.13 (s, 1H), 1.40 (d, *J* = 7.2 Hz, 3H). ^13^C NMR (150 MHz, acetone-*d*_6_) *δ* 175.36, 157.37, 131.85, 129.12, 116.30, 115.48, 108.61, 86.28, 81.86, 75.30, 75.25, 43.24, 16.40. HRESIMS *m/z* 295.0816 (calcd for C_14_H_16_O_7_ [M − H]^−^,295.0823).

**10** (42 %), colorless oil, main isomer: ^1^H NMR (600 MHz, acetone-*d*_6_) *δ* 7.29 (d, *J* = 8.8 Hz, 2H), 6.82 (d, *J* = 8.8 Hz, 2H), 5.69 (s, 1H), 4.70 (s, 1H), 4.39 (d, *J* = 6.1 Hz, 1H), 4.32 (dd, *J* = 5.0, 3.1 Hz, 1H), 4.05–4.08 (m, overlaped, 1H), 3.97 (dd, *J* = 11.5, 3.0 Hz, 1H), 3.83 (s, 1H), 3.77 (s, 3H), 3.23 (q, *J* = 7.3 Hz, 1H), 1.51 (d, *J* = 7.3 Hz, 3H). ^13^C NMR (150 MHz, acetone-*d*_6_) *δ* 176.34, 159.64, 131.76, 130.42, 114.69, 113.74, 109.15, 87.03, 86.29, 75.31, 75.10, 55.41, 43.25, 16.39. HRESIMS *m/z* 333.0949 (calcd for C_15_H_18_O_7_ [M + Na]^+^,333.0945).

**11** (78 %), colorless oil: ^1^H NMR (600 MHz, acetone-*d*_6_) *δ* 7.57 (s, 1H), 6.92 (d, *J* = 1.9 Hz, 1H), 6.80–6.67 (m, 2H), 5.87 (s, 1H), 4.69 (s, 1H), 4.51 (s, 1H), 4.31 (m, 1H), 4.09 (dd, *J* = 9.8, 5.4 Hz, 1H), 4.01 (dd, *J* = 9.7, 3.0 Hz, 1H), 3.82 (s, 1H), 3.78 (s, 3H), 3.11 (d, *J* = 13.5 Hz, 1H), 2.94 (d, *J* = 13.5 Hz, 1H). ^13^C NMR (150 MHz, acetone-*d*_6_) *δ* 175.94, 147.61, 146.55, 126.16, 124.37, 115.33, 115.21, 108.30, 86.97, 80.79, 75.48, 75.34, 56.14, 41.03. HRESIMS *m/z* 335.0742 (calcd for C_14_H_16_O_8_ [M + Na]^+^,335.0737).

**12** (58 %), white foam: ^1^H NMR (600 MHz, acetone-*d*_6_) *δ* 8.22 (s, 1H), 7.05 (d, *J* = 1.9 Hz, 1H), 6.92 (d, *J* = 1.8 Hz, 1H), 5.93 (s, 1H), 4.73 (s, 1H), 4.60 (s, 1H), 4.35 (dd, *J* = 5.2, 3.3 Hz, 1H), 4.11 (dd, *J* = 9.8, 5.5 Hz, 1H), 4.03 (dd, *J* = 9.8, 3.0 Hz, 2H), 3.81 (s, 3H), 3.07 (d, *J* = 13.8 Hz, 1H), 2.97 (m, overlaped, 1H). ^13^C NMR (150 MHz, acetone-*d*_6_) *δ* 175.50, 148.28, 143.97, 127.58, 127.50, 114.28, 108.59, 108.29, 87.26, 80.38, 75.67, 75.38, 56.61, 40.46. HRESIMS *m/z* 388.9870, 390.9851 (M + 2-H) (calcd for C_14_H_15_BrO_8_ [M – H]^−^, 388.9878).

Method C [27] for lactone **16**: To l-AA (2.3 eq.) in 10 mL phosphate-citrate buffer (PH 5.0) was added corresponding alcohol **B11** (1 mmol, 1 eq.) in 1 mL EtOH, and the solution stirred at room temperature for 8 h. The reaction mixture was extracted with EtOAc, the extract was washed with water, dried over Na_2_SO_4_ (s), and the solvent was evaporated under reduced pressure and the residue was purified by column chromatography with DCM/MeOH (10/1) to afford lactone **16** (57 %) a gray foam. ^1^H NMR (600 MHz, acetone-*d*_6_) *δ* 10.18 (s, 1H), 7.66 (d, *J* = 8.0 Hz, 1H), 7.37 (d, *J* = 8.0 Hz, 1H), 7.29 (d, *J* = 2.3 Hz, 1H), 7.07 (t, *J* = 8.0, 1.0 Hz, 1H), 6.98 (t, *J* = 8.0, 1.0 Hz, 1H), 5.92 (s, 1H), 4.65 (s, 1H), 4.50 (s, 1H), 4.28 (m, 1H), 4.10 (dd, *J* = 9.7, 5.5 Hz, 1H), 4.01 (dd, *J* = 9.7, 3.2 Hz, 1H), 3.91 (s, 1H), 3.39 (d, *J* = 14.3 Hz, 1H), 3.25 (d, *J* = 14.3 Hz, 1H). ^13^C NMR (150 MHz, acetone-*d*_6_) *δ* 176.47, 137.13, 129.06, 126.46, 122.00, 120.06, 119.52, 111.99, 108.53, 107.89, 87.24, 80.22, 75.47, 75.45, 31.22. HRESIMS *m/z* 328.0795 (calcd for C_15_H_15_NO_6_ [M + Na]^+^, 328.0792).
